# In-vivo toxicokinetics of chromium in human blood and urine after intravenous injection of chromate

**DOI:** 10.1007/s00204-025-04247-1

**Published:** 2025-12-08

**Authors:** Sonja Kilo, Anna Wolfschmidt, Florian Tobias Nickel, Andrea Kaifie, Thomas Göen, Hans Drexler

**Affiliations:** 1 Institute and Outpatient Clinic of Occupational, Social, and Environmental Medicine, Faculty of Medicine, Friedrich-Alexander-Universität Erlangen-Nürnberg (FAU), Henkestraße 9–11, 91054 Erlangen, Germany; 2 Department of Neurology, Universitätsklinikum Erlangen, Friedrich- Alexander-Universität Erlangen-Nürnberg, (FAU), Schwabachanlage 6, 91054 Erlangen, Germany

**Keywords:** Chromium, Toxicokinetics, Exposure assessment, Biomonitoring, Occupational medicine, Environmental medicine.

## Abstract

Although intoxication with hexavalent chromium [Cr(VI)] compounds can be fatal to humans, reliable data on chromium toxicokinetics are largely missing. We report on a rare case of intravenous Cr(VI) poisoning and its impact on the scientific understanding of chromium toxicokinetics and red blood cells’ (RBCs’) lifespan in the human body. The approximate amount of injected chromium was between 0.33 and 0.66 g. Laboratory findings were collected over a half-year period from the time of injection. We monitored kidney function, liver function, RBC parameters, and chromium concentration in RBCs, plasma, and urine. In all samples, the total chromium concentration was quantified by inductively coupled plasma-mass spectrometry. By injecting Cr(VI) into his vein, the patient inadvertently labeled all his RBCs with chromium. This gave us the unique opportunity to calculate the RBC lifespan rather than just to estimate it. Based on RBC breakdown rates, the average lifespan was calculated to be 111 days, and the maximum lifespan to be 141.4 days. At about 24 weeks post-injection, the RBC chromium concentration approached background-exposure values, whereas chromium in plasma reached a plateau considerably higher than the reference value. These results confirm that there is a long-term storage compartment for chromium in the human body, which releases chromium into plasma but leaves the RBC chromium concentration unaffected. Therefore, this singular case of Cr(VI) poisoning provides us with the long-awaited scientific proof that chromium in the RBC fraction is a specific monitoring parameter for exposure to the carcinogen Cr(VI) in occupational and environmental settings.

## Introduction

Hexavalent chromium compounds contain the element chromium in its + 6 oxidation state. They can be absorbed into the body via oral ingestion, inhalation, or permeation through the skin. Both intracellularly and extracellularly, the strongly oxidizing hexavalent chromium (Cr(VI)) is rapidly reduced to trivalent chromium (Cr(III)) by ascorbate and glutathione. This process releases radical oxygen and sulfur species, which can cause toxic cell death or interfere with various cell structures, such as enzymes and DNA. Cr(VI) compounds are therefore not only carcinogenic but also acutely toxic to humans (Hartwig et al. 2016). The lethal oral dose for soluble chromates can be reached at concentrations between 50 and 70 mg/kg body weight, according to the World Health Organization (WHO 1988). Acute signs and symptoms of intoxication include nausea, vomiting, spasms, diarrhea, and hemorrhaging in the kidneys and gastrointestinal tract, which can lead to cardiovascular shock. Liver necrosis may be observed as a post-acute effect (Hartwig et al. 2016, WHO 1988).

Occupational or environmental exposure to chromium can be monitored by using the parameter chromium in urine. However, the parameter chromium in urine is non-specific: it is not only influenced by the carcinogenic Cr(VI) compounds, but also by other non-carcinogenic chromium compounds like Cr(III). It has been proposed that exposure to the carcinogen Cr(VI) can be specifically monitored by determining chromium in the red blood cell (RBC) fraction of whole blood (MAK Commission 2024), but although this parameter is widely used, the presented case is the first case in humans in which this could be validated.

Information on the distribution of hexavalent chromium in the human body and its elimination is limited and largely based on animal experiments and observational studies of occupationally exposed workers (Hartwig et al. 2016), which is in clear contrast to the industrial and environmental importance of chromium. It is widely used throughout numerous industrial sectors and, as an environmental toxin, it poses a major global public health problem and has become an increasing environmental burden. Only very few studies have examined subjects who ingested a small amount of a Cr(VI) compound (Kuykendall et al. 1996), but there have been no studies in which Cr(VI) was administered intravenously. Case reports of poisoning with Cr(VI) date back nearly as far as its industrial use (Lehmann 1914), but the usual route of exposure is inhalation or ingestion, and almost all case reports of deliberate Cr(VI) intake describe the ingestion of a Cr(VI) compound with suicidal intent.

At *Universitätsklinikum Erlangen*, we had the rare opportunity to observe and follow a case of intentional intravenous intoxication with Cr(VI), which the patient survived. We supervised the case for almost half a year from the time of injection and obtained insights into the clinical effects and toxicokinetics of systemically absorbed Cr(VI). Apart from illustrating the emergency treatment and case management of this unique type of intoxication, we were able to draw important lessons from this case both in terms of RBC kinetics and best practices for assessing exposure scenarios involving Cr(VI).

## Methods

For the analysis of chromium in RBCs, plasma, and urine, blood was collected in EDTA-containing Monovette^®^ tubes (Sarstedt, Nümbrecht, Germany) and centrifuged to separate plasma and RBCs. In view of the extremely high chromium levels in our patient, the use of EDTA Monovettes^®^ for blood sampling can be considered unproblematic, as possible contamination from the sampling method is negligible compared to the actual values. Urine samples were collected in standard urine cups, drawn into urine Monovettes^®^ (Sarstedt, Nümbrecht, Germany), and then sent to the laboratory. The RBC fraction was washed three times with saline and resuspended to the initial volume in double-distilled water to destroy the RBC membrane and to release the intracellular chromium content.

The first urine and blood samples were analyzed for chromium content by the Department of Toxicology at the Technical University of Munich, Germany. All other samples were analyzed in the laboratory of the Institute and Outpatient Clinic of Occupational, Social, and Environmental Medicine at Friedrich-Alexander-Universität Erlangen-Nürnberg. Total chromium concentration in serum, RBCs, and urine was quantified by inductively coupled plasma-mass spectrometry (ICP-MS) with collision cell (Agilent 7500cx). For ICP-MS plasma generation, we used Argon of 99.996% purity (AirLiquide). Helium (99.999% purity, AirLiquide) served as collision gas for the background reduction carried out for chromium. Prior to ICP-MS, all blood and urine samples were subjected to acidic thermic digestion. For this purpose, blood and urine samples were diluted 1:10 with a 0.2% nitric acid solution. Calibration standards were prepared in matrix blanks and spiked with incremental quantities of chromium. During ICP-MS analysis, the ion mass 52 was monitored to quantify chromium content. In each series, we performed quality controls by analyzing control material (spiked matrix blanks) and assessing the results by quality control chart analysis.

A more detailed description of the analytical procedure is available in publications by Nübler et al. (2022) and Fortmann et al. (2017). The accuracy of the analytical procedure was verified by successful participation in the quality-assurance program of HBM4EU as well as the German External Quality Assessment Scheme (G-EQUAS) (Nübler et al. 2022; Göen et al. 2012).

Data processing and analysis was performed with IBM SPSS Statistics 25.0 and Origin 2015.

## Observations and results

### Intravenous Cr(VI) intoxication and case management

A 29-year-old patient who was admitted to the emergency room at the university hospital in Erlangen (*Universitätsklinikum Erlangen*) reported the injection of a saturated solution of Cr(VI)oxide (chromium trioxide) into his left cubital vein in suicidal intent. Extreme pain at the injection site had led him to interrupt the injection and call an ambulance.

The rescue team documented livid discoloration in the patient’s lower arms and an indurated cubital vein. Upon admission to the hospital (~ 3.5 h post-injection), blood tests showed a positive result for cannabinoids and extremely elevated lipase activity (2752 U/l). Following consultation with the poison center, continuous veno-venous hemodialysis (CVVHD), combined with volume replacement and acetylcysteine therapy, was initiated.

**Fig. 1:**
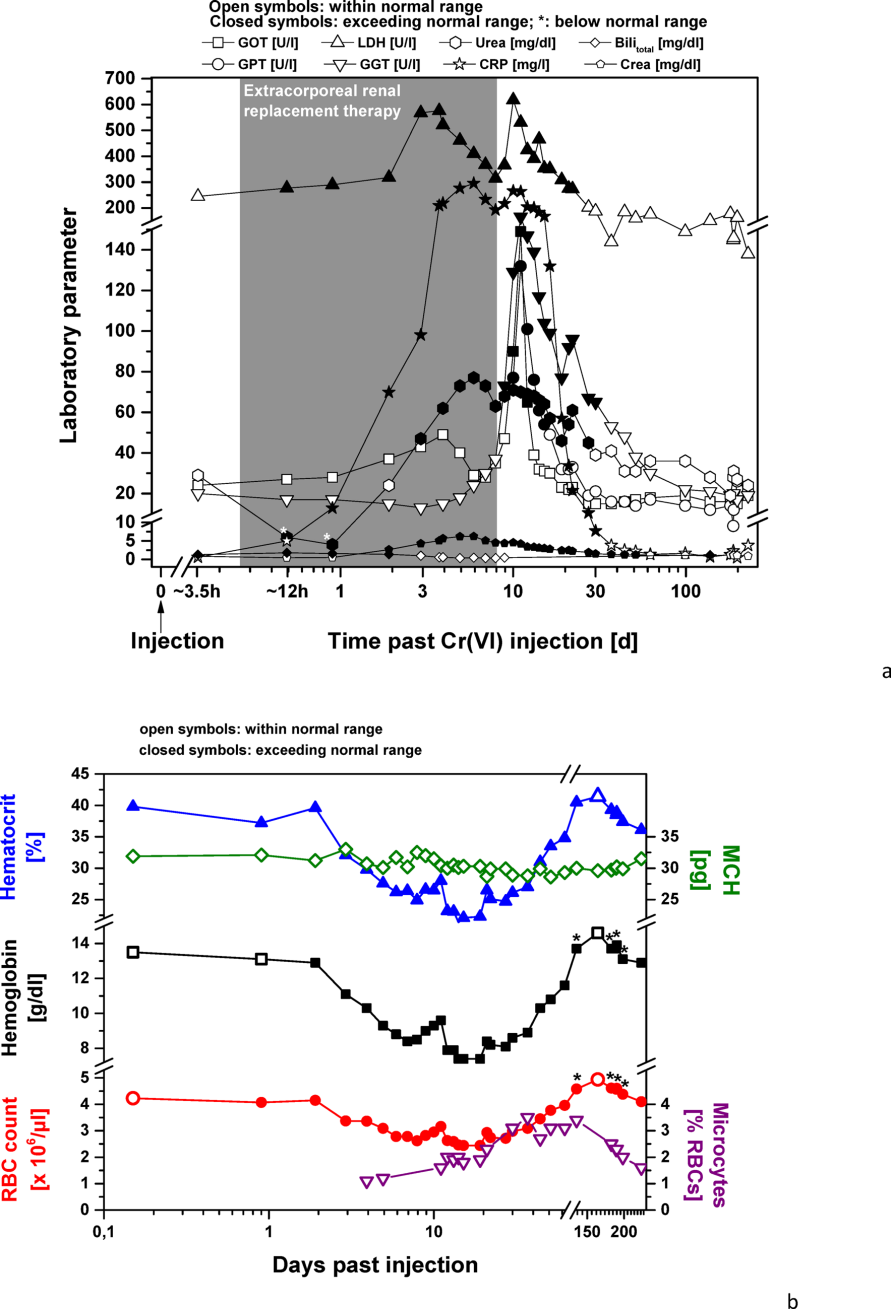
**a** Changes in selected laboratory parameters of liver function (GOT; GPT; LDH; GGT), kidney function (urea; crea), hemolysis (total Bili; GOT; LDH), and the acute-phase protein CRP following injection of chromium trioxide. Values within the normal range are depicted by open symbols. Values exceeding the normal range are represented by closed symbols; if below the normal range, closed symbols are asterisked. ** b** Changes in selected RBC parameters (count; Hb; Hct; MCH; and microcytes). Values within the normal range are depicted by open symbols. Values below the normal range are represented by closed symbols. Slightly varying normal ranges were given by the different laboratories processing the routine blood work. As a result, values just within the reference range in one laboratory could be just outside the reference range of another laboratory. Those values are asterisked

A few hours after the injection, the patient complained of abdominal pain and diarrhea, which lasted for about 2 days. He began to develop anemia with increased hemolytic markers, the latter normalizing within the second day. Due to the presence of metabolic acidosis and an acute kidney injury (AKIN) (Stage 3), a substitution therapy and forced diuresis were initiated in addition to dialysis. GOT (serum glutamic oxaloacetic transaminase = aspartate transaminase) and GGT (gamma-glutamyltransferase) stayed within the normal range, while GPT (serum glutamic pyruvic transaminase = alanine transaminase) levels started to rise on Day 3. The patient described an impaired sense of taste, which lasted for 1–2 weeks. Following termination of renal replacement therapy, the patient was transferred to the psychiatric department of *Universitätsklinikum Erlangen* on Day 9. After a peak around Day 10–11, most laboratory values returned to their respective normal ranges within the next 3 weeks. Liver-function parameters initially increased in the following order: GGT > GOT > GPT. GGT levels remained elevated for 3 weeks, GPT for 1 week, and GOT for 3 days. Kidney function recovered within the same time frame. Blood coagulation, hemolytic markers, and C-reactive protein (CRP) also normalized during this period (Fig. 1a and b).

### Changes in hemogram

The increase in bilirubin within 3.5 h of the injection of Cr(VI) was indicative of intravascular hemolysis caused by Cr(VI) intoxication. Acute hemolysis lasted for a maximum of 2.5 days, since bilirubin values returned to normal in less than 3 days. At about that time, the RBC count and the Hb started to drop substantially. Apart from the chromium-related RBC breakdown, this drop was probably due to a dilution effect from volume-replacement therapy and a loss of RBCs during CVVHD on Days 1–8. Afterwards, other factors–like an adverse effect of the RBCs’ chromium load–may have caused a further decrease in these parameters. About three weeks after the incident, RBC count and hemoglobin had reached their lowest levels and started to rise again, whereas the hemoglobin concentration somewhat lagged.

It took about 17 weeks (120 days) to reach initial values (as taken 3.5 h post-injection), which then further increased to 115% of the initial RBC count and 108% of the initial Hb concentration in the following 3.5 weeks. Since the first measurement was taken in the acute phase, it is likely that the values were already low at that time and therefore appeared elevated later.

### Chromium in blood and urine

Based on the patient’s description of the preparation of the Cr(VI) solution, it can be calculated that he injected himself with about 1–2 ml of a saturated Cr(VI)-oxide solution intravenously. Since the solubility of Cr(VI)O_3_ is 0.63 g/ml and chromium accounts for ~ 52% of the weight of the oxide, the amount of injected chromium was between 0.33 and 0.66 g. Taking into account the patient’s body weight of 60 kg and his height of 173 cm, his estimated total blood volume was 4435 ml, according to the Nadler equation (Nadler et al. 1962), which adds up to an estimated amount of 0.074–0.148 g chromium per liter blood.

**Fig. 2:**
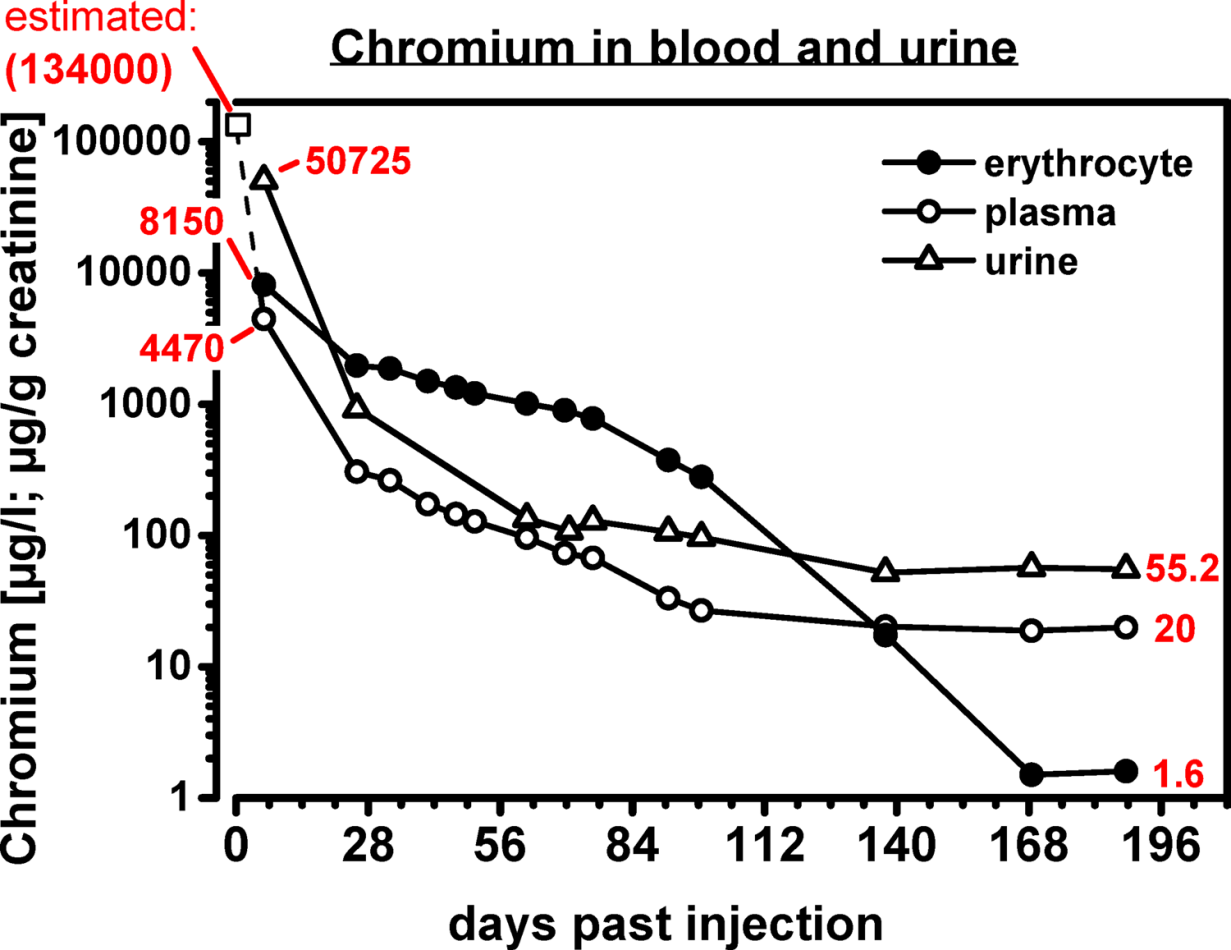
Changes in chromium concentration in RBCs, plasma, and urine following intentional intravenous injection of hexavalent chromium

Starting from this range, values dropped within 6 days to 6.52 mg/l in blood, 8.15 mg/l in RBCs, and 4.47 mg/l in serum. At the same time, 35 mg chromium/l were excreted in urine at a glomerular filtration rate of 11 ml/min. Over the following weeks, the chromium concentration decreased exponentially in all tested media (Fig. 2).

### Calculation of RBC lifespan

**Fig. 3:**
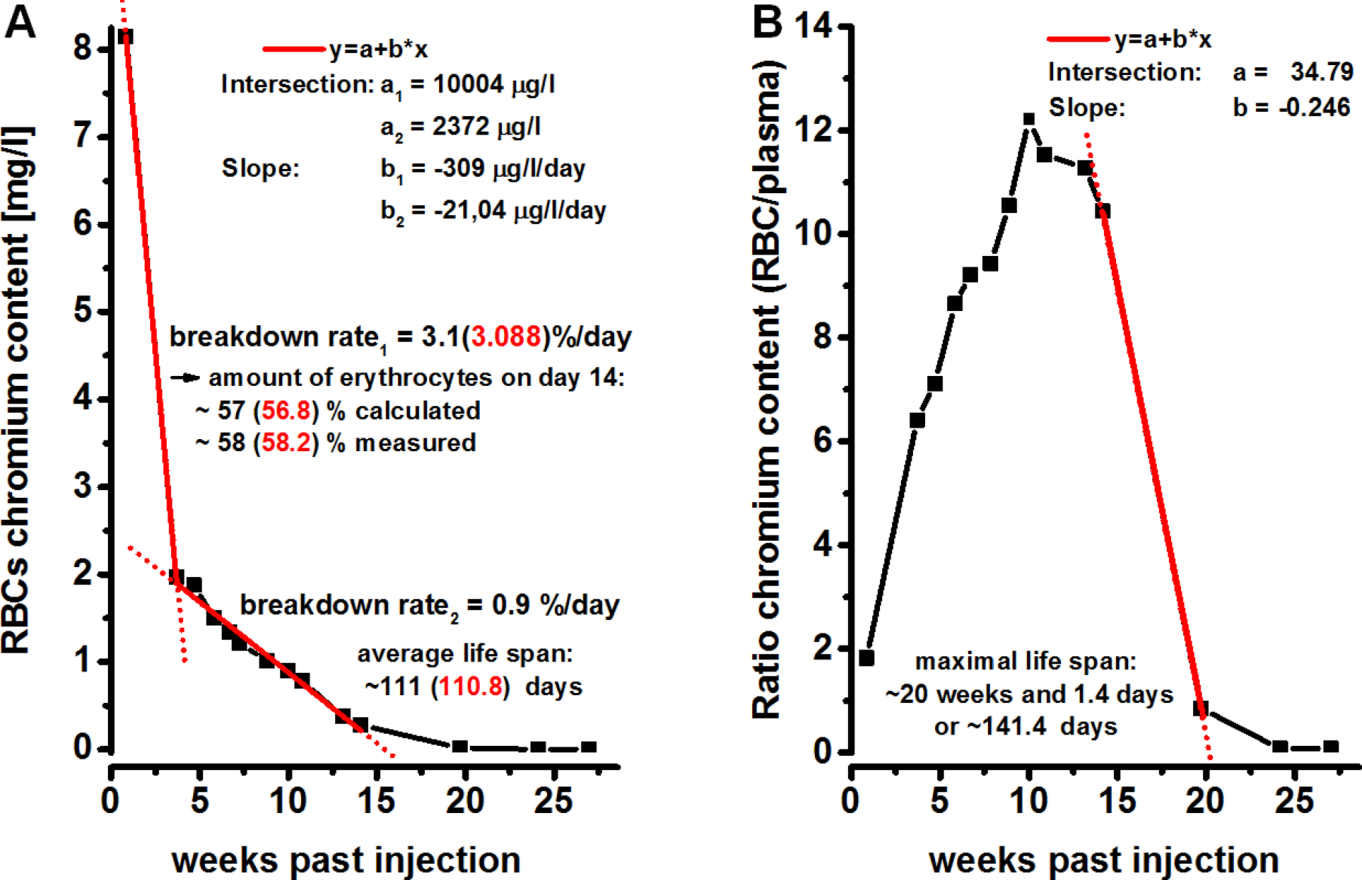
shows the calculated lifespan of RBCs. The average RBC lifespan of about 111 days is shown in (**A**) and the maximum RBC lifespan of about 141.4 days is shown in (**B**)

Following a period with a very steep decline, which corresponds to an RBC breakdown rate of 3.1% per day, the decline in RBC chromium content leveled off, resulting in an RBC breakdown rate of about 0.9% a day. Based on this breakdown rate, the average lifespan of the patient’s RBCs is calculated to be approximately 111 days (Fig. 3A). RBC chromium content leveled off around Week 24 post-injection, reaching a final concentration of 1.6 µg/L, which corresponds to the upper range of background exposure values (Lewalter et al. 1991; Santonen et al. 2022). At post-injection Day 111 ( ≙ calculated average RBC lifespan), the ratio of chromium content of RBCs vs. plasma was still elevated (7.5), but was determined to be in a phase of steep decline, yielding a maximum RBC lifespan of about 141.4 days (Fig. 3B). At later points in time, the ratio of chromium content of RBCs vs. plasma reached and remained at 0.08, indicating a balanced chromium distribution between RBCs and plasma.

## Discussion

The specific uptake pathway, namely intravenous injection rather than oral ingestion, could be the reason why the patient survived this high dose of Cr(VI). Bypassing the first-pass route to the liver and the rapid chromium uptake into RBCs may have prevented adverse liver effects, since no fatal liver injuries with complete liver failure occurred.

### Changes in hemogram

The blood-test results in our case report indicate that the first three days were characterized by a hemolytic loss of RBCs. Chromium can interact directly with the RBC membrane, with Cr(III) having a stronger effect on membrane stability than Cr(VI) (Kitagawa et al. 1982; Lupescu et al. 2012; Suwalsky et al. 2008). It seems plausible that the rapid increase in both intra-erythrocytic and extra-erythrocytic Cr(III) could have triggered hemolysis. Furthermore, CVVHD treatment during this period may have impaired RBC integrity.

Different mechanisms could explain why the initial rapid decline of about 4 weeks went far beyond the state of acute hemolysis. The loss of chromium during this phase, which was higher by a factor of 3.5, might be due to distribution processes of chromium into other tissues. Cr(III) either cannot cross biological membranes or crosses them only very slowly, such as after having formed complexes with biomolecules which can interact with the cell membrane (Kortenkamp et al. 2008). Recent studies suggest cellular uptake via endocytosis after formation of a Cr(III)-transferrin/transferrin-receptor complex and describe transport by a low-molecular-weight chromium-binding substance (LMWCr) from the cells to the bloodstream (Bergant et al. 2020; Vincent and Love 2012; Levina et al. 2016; Bonvin et al. 2017; Edwards et al. 2021; Clodfelder et al. 2005). The initial rapid decline in chromium could also be the result of an increase in eryptosis of RBCs more susceptible to Cr(VI) effects or with a higher initial load of Cr(VI).

### RBC lifespan

The calculated average RBC lifespan of 111 days is within the expected average RBC lifespan of 100 to 120 days. These values were determined based on cohort- or random-labeling methods (Garby et al. 1971, Mock et al. 1999). In cohort labeling, substances needed during hematopoiesis, like nitrogen or iron, are (partially) substituted by another isotope whose degradation is observed. However, the uncertain time at which these isotopes are incorporated into RBC precursor cells can result in an overestimation of RBC lifespan. In the case of random labeling, RBCs are collected from a subject, incubated for a certain time with a labeled substance, and reinjected. For this method, chromium-51 is still the “gold standard” (Shrestha et al. 2016), but the ex-vivo handling of RBCs could compromise RBC integrity, especially in older RBCs, which could also lead to an overestimation of RBC lifespan.

Data on the maximum lifespan of RBCs is sparse, but is thought to be about 21 weeks (Quinlivan et al. 2008). In silico, RBC survival estimates are in the range of 150–160 days for maximum lifespan (Shrestha et al. 2016). With about 141 days, the calculated maximum lifespan of the patient’s RBCs was slightly shorter, but within the range expected from the literature.

### Distribution of chromium in the human body

At around Week 20–24 post-injection, the RBC chromium content leveled off and reached a concentration consistent with the RBC chromium concentrations found in samples of workers without occupational exposure to chromium (Santonen et al. 2022). Chromium in plasma reached a plateau of 20 µg/L around 20 weeks after exposure, which is considerably higher than the reference value (95th percentile) in the general population of about 0.1 µg/L (Heitland et al. 2021). These results are consistent with the assumption that chromium may be released into the bloodstream from storage compartments over a very long period post-exposure (Thomann et al. 1994; Welinder et al. 1983). As the RBC chromium concentration approached background-exposure values about 24 weeks after the intentional Cr(VI) injection, it can be assumed that Cr(III)–but not Cr(VI)–is released form these compartments.

The parameter chromium in urine shows a clear correlation with chromium levels in plasma. After a sharp decline in the first three months after exposure, the amount of chromium in urine also plateaued at around the 20th week at a significantly elevated level compared to the reference value (55.2 µg/g creatinine vs. 0.43 µg/g creatinine; Heitland et al. 2021). There are no studies available that present data on chromium half-lives and the elimination of chromium over time in urine or plasma after high chromate exposure. However, our hypothesis that the chromium plasma plateau (and the associated chromium urine plateau) results from a slow, gradual elimination of chromium from storage compartments in the human body is supported by data provided by Tandon et al. (1977). In Tandon’s study, electroplating workers tended to have higher chromate excretion in their urine the longer they had worked at the respective workplaces.

## Conclusion

Four conclusions can be drawn from this singular case. First, the mean lifespan of RBCs appears to be stable within humans, even after chemically induced trauma.

Second, the maximum RBC lifespan is about one month longer than their average lifespan and was calculated to be 141.4 days. Since the patient virtually labeled all his RBCs at once, the amount of chromium in the RBCs was still detectable, with most of the originally labeled RBCs already removed from circulation by senescence, even 20 weeks later, allowing for the calculation rather than the estimation of maximum lifespan.

Third, the elevated plasma chromium level, which was still stable 20 weeks after the Cr(VI) injection, supports the theory of a long-term storage compartment for chromium in the human body from which it can be released.

Fourth, the results prove the applicability of chromium in the RBC fraction as a biomonitoring parameter for Cr(VI) uptake up to 20 weeks after occupational or environmental exposure. In contrast, the applicability of chromium in plasma for risk assessment is questionable due to its complex kinetics from inter-compartment chromium exchange.

## Data Availability

The datasets generated and analysed during the current case study are available from the corresponding author on reasonable request. Correspondence and requests for materials should be addressed to Dr. Anna Wolfschmidt, Institute and Outpatient Clinic of Occupational, Social and Environmental Medicine, Friedrich-Alexander-Universität Erlangen-Nürnberg, Henkestr. 9–11, 91054 Erlangen; anna.wolfschmidt@fau.de.

## Abbreviations

AKIN: Acute kidney injury network classification
aPTT: Activated partial thromboplastin time
AT III: Antithrombin
BIL: Indirect bilirubin
CHE: Cholinesterase enzyme
Crea: Serum creatinine level
Cr: Chromium
CVVHD: Continuous veno-venous hemodialysis
GFR: Glomerular filtration rate
GGT: Gamma-glutamyltransferase
GOT: Glutamic oxaloacetic transaminase = aspartate aminotransferase (AST)
Hb: Hemoglobin concentration
Hct: Hematocrit
LDH: Lactate dehydrogenase
MCH: Mean corpuscular hemoglobin content
MPV: Mean platelet volume
PT: Prothrombin time (Quick-Test)
RBC: Red blood cell
RDW: Red-cell distribution width
TBV: Total blood volume
TT: Thrombin time
